# The Role of Bone Mineral Density in a Successful Lumbar Interbody Fusion: A Narrative Review

**DOI:** 10.7759/cureus.54727

**Published:** 2024-02-22

**Authors:** Kyriakos N Bekas, Christos Zafeiris

**Affiliations:** 1 Orthopaedics, 1st Orthopaedics Department, G. Gennimatas General Hospital, Athens, GRC; 2 Th. Garofalidis Laboratory for Research of the Musculoskeletal System, Medical School, National and Kapodistrian University of Athens, Athens, GRC; 3 Orthopaedics and Spine Surgery, Metropolitan General Hospital, Athens, GRC

**Keywords:** mineral bone metabolism, osteoporosis treatment, lumbar interbody fusion, s: osteoporosis, cage subsidence, bone mineral density

## Abstract

Background: The incidence of osteoporosis is a prime concern, especially in parts of the world where the population is aging, such as Europe or the US. Many new therapy strategies have been described to enhance bone healing. Lumbar interbody fusion (LIF) is a surgical procedure that aims to stabilize the lumbar spine by fusing two or more vertebrae using an interbody cage. LIF is a standard treatment for various spinal conditions, such as degenerative disc disease, spinal stenosis, and spondylolisthesis. However, successful fusion is challenging for patients with osteoporosis due to their reduced bone mineral density (BMD) and increased risk of cage subsidence, which can lead to implant failure and poor clinical outcomes.

Methods:* *A comprehensive literature search yielded 220 articles, with 16 ultimately included. Keywords included BMD, cage subsidence, osteoporosis, teriparatide, and lumbar interbody fusion.

Results: This review examines the relationship between BMD and LIF success, emphasizing the importance of adequate bone quality for successful fusion. Preoperative assessment methods for BMD and the impact of low BMD on fusion rates and patient outcomes are discussed. Additionally, techniques to improve fusion success in patients with weakened bone density, such as biological enhancement and BMD-matched interbody cages, are explored. However, consensus on the exact BMD threshold for a successful outcome remains elusive.

Conclusion: While an apparent correlation between BMD and fusion rate in LIF procedures is acknowledged, conclusive evidence regarding the precise BMD threshold indicative of an increased risk of unfavorable outcomes remains elusive. Surgeons are advised to exercise caution in surgical planning and follow-up for patients with lower BMD. Furthermore, future research initiatives, particularly longitudinal studies, are encouraged to prioritize the examination of BMD as a fundamental risk factor, addressing gaps in the literature.

## Introduction and background

Osteoporosis is a condition in which bone mass is reduced, bone architecture deteriorates, and ultimately leads to an increased risk of fragility fractures. Bones become porous and brittle, resulting in a higher susceptibility to fragility fractures. The prevalence of osteoporosis increases worldwide, resulting in 200 million osteoporotic fractures yearly [1].

Osteoporosis can affect any bone in the body. Still, the main clinical manifestations include the so-called "osteoporotic fractures," mostly involving the spine, the proximal femur, and the distal radius. Vertebral compression fractures are a common consequence of osteoporosis, although they can also occur due to other factors such as trauma or certain medical conditions. They usually occur without symptoms and can be found incidentally on imaging for other, unrelated pathologies [2]. Vertebral compression fractures refer to the compression or collapse of a vertebral body in the spine. These fractures often affect vertebrae characterized by reduced bone mineral density (BMD) and are associated with high rates of morbidity, height loss, kyphosis, restrictive lung disease, chronic back pain, and, eventually, functional impairment. Although there are very effective treatments to reduce fracture risk, it seems that only 30% of patients with fragility fractures underwent a BMD evaluation test or examination [2].

BMD is a critical factor in diagnosing, managing, and treating osteoporosis, as it measures the amount of minerals (mainly calcium and phosphorus) in bone tissue. BMD testing is used to diagnose osteoporosis and assess the risk of fractures. The most commonly used method to measure BMD is dual-energy X-ray absorptiometry (DEXA) scanning, which can detect minor bone mass and density changes. The World Health Organization (WHO) defines osteoporosis based on BMD measurements. DEXA scores are reported as "T-scores" and "Z-scores." The T-score compares a person's bone density with that of a healthy 30-year-old of the same sex. The Z-score compares a person's bone density with that of an average person of the same age and sex. A patient with osteoporosis has a BMD T-score of -2.5 or lower, and that person is at a high risk of fractures. A T-score of -1.0 to -2.5 indicates osteopenia, meaning below-normal bone density without full osteoporosis [3]. BMD is also used to monitor the effectiveness of osteoporosis treatments. Osteoporosis medications aim to increase BMD and reduce the risk of fractures, although the effects may take months or years to become noticeable.

Lumbar interbody fusion (LIF) is a surgical procedure that aims to stabilize the lumbar spine by fusing two or more vertebrae using an interbody cage. LIF is a standard treatment for various spinal conditions, such as trauma, degenerative disc disease, spinal stenosis, spondylodiscitis, and spondylolisthesis. However, successful fusion is challenging in patients with osteoporosis, as BMD has been associated with postoperative complications such as cage subsidence [4-10], pedicle screw loosening [11,12], and subsequent adjacent fractures [13,14].

LIF surgical techniques’ ability to restore disc height, indirectly decompress the nerves, and preserve the anterior and posterior stabilizing elements are the main reasons why the preference among spine surgeons is growing worldwide. Five main LIF approaches are described in the literature: anterior lumbar interbody fusion (ALIF), posterior lumbar interbody fusion (PLIF), transforaminal lumbar interbody fusion (TLIF or MI-TLIF), lateral lumbar interbody fusion (LLIF), and oblique lumbar interbody fusion/anterior to the psoas (OLIF/ATP) [15].

This study aims to review the literature and investigate the effect of BMD on the success of LIF in osteoporotic patients. The association between BMD and perioperative and postoperative complications will be examined. The findings of this study can contribute to the development of evidence-based guidelines for the management of osteoporotic patients undergoing LIF and ultimately improve their clinical outcomes.

## Review

Search methodology

An extensive search was conducted in Web of Science (WoS), PubMed, Scopus, and Cochrane from 1979 to 2024 to investigate the role of BMD in a successful LIF in osteoporotic patients. The keywords mineral bone metabolism, osteoporosis treatment, lumbar interbody fusion, osteoporosis, and cage subsidence were used after an initial screening yielded 225 studies; 52 that met our inclusion criteria were identified and included.

A total of 225 potentially relevant studies were identified during the database search, of which 70 were duplicates and removed. Of the remaining 155 studies, 87 were excluded because they dealt with irrelevant aspects of the correlation between BMD and LIF. Out of the 68 articles selected for reading, only 52 met the criteria of this literature review.

Case reports, case series, systematic reviews, and meta-analyses concerning patients with osteoporosis diagnosed and treated with LIF procedures, as well as research on LIF enhancement techniques and pharmaceutical, biological, and biomechanical enhancement of low BMD, met the inclusion criteria.

The exclusion criteria were surgical techniques, animal studies, lack of direct relevance to osteoporosis or BMD, and studies with insufficient, incomplete, or irrelevant data.

Data analysis

A summary of the included studies is seen in Table 1 [4,12,16-65]. The included studies were divided based on the success of spinal fusion and its relationship with BMD and pharmacological and biomechanical enhancement.

**Table 1:** Summary of the studies included Source: References [4,12,16–65]

Focus of Study	Summary	Studies
BMD Significance in LIF	In this section, the significance of bone mineral density (BMD) in lumbar interbody fusion (LIF) is discussed. It explores how fusion is facilitated by adequate BMD, along with the preoperative assessment methods and the implications of low BMD on fusion rates and clinical outcomes.	Cho et al. [4], Halvorson et al. [12], Oh et al. [16], Falowski et al. [17], Au et al. [18,26], Pennington et al. [19], Reitman et al. [20], Coe et al. [21], Bjerke et al. [22], Matsukawa et al. [23], Carter et al. [24], Marshall et al. [25], Hsu et al. [27], Choy et al. [28], Collino et al. [29], Murr et al. [60], Rezvani et al. [61]
Pharmacological and Biological Enhancement in LIF	In this part, various biological enhancement strategies aimed at improving BMD in patients undergoing LIF are examined. Interventions such as bisphosphonates, teriparatide therapy, and biologics are discussed, along with their role in optimizing fusion success in individuals with compromised BMD.	Bisphosphonates [31,32,36–41], Teriparatide [32–34,42–46], Vitamin D [35,47], Denosumab [22,32,48–51], Romosozumab [52–54]
Biomechanical Enhancement in LIF	Here, the focus is placed on biomechanical enhancement techniques employed to tackle BMD-related challenges in LIF. The use of BMD-matched interbody cages, surgical approaches, and other biomechanical considerations aimed at improving fusion outcomes in patients with reduced BMD are explored.	Falowski et al. [17], Weng et al. [55], Kivell et al. [56], Wang et al. [57], Deligianni et al. [58], Liu et al. [59], Rezvani et al. [61], Tabarestani et al. [62], Mahmoodkhani et al. [63], Mo et al. [64], Tavares et al. [65]

BMD importance in lumbar interbody fusion

LIF is a surgical procedure in which an intervertebral disc is removed and replaced with a bone graft or interbody device to promote spinal fusion. For the fusion to be successful, there must be adequate bone quality and quantity to support the implanted graft or device, and BMD plays a critical role in this process.

In the context of LIF, BMD is significant for several reasons. BMD is currently the most accurate proxy for bone strength. One of the primary reasons BMD is significant in LIF is its impact on implant success. A screw's purchase, instrumentation failure, and the likelihood of developing pseudoarthrosis, adjacent segment disease, or junctional kyphosis are all significantly influenced by bone density during an instrumented fusion procedure [19]. Implants used in LIF surgeries, such as cages, bone grafts, or interbody devices, rely on the surrounding bone tissue for support and stability. Underlying low BMD has been associated with severe post-operative, device-related complications such as cage subsidence, pedicle screw loosening, subsequent adjacent-level fractures, and the necessity of revision surgery [17]. Studies conducted in vitro have shown a linear correlation between screw pullout force and DXA-measured BMD [12,20,21]. In patients with lower BMD, the bone tissue may be weaker and unable to support the implant, increasing the risk of implant failure, such as subsidence or migration [22,23]. In addition, lower BMD can result in a decreased fusion rate, as the bone may be unable to integrate with the implanted graft or device. Compressive bone strength and trabecular bone density are highly correlated [24] and DXA-measured spine BMD has been shown in clinical studies to be negatively correlated with the risk of compression fractures [25]. This can result in pseudarthrosis (failure of bone to fuse) and persistent pain or instability. Bioactive or biokinetic implants are currently being designed and produced to reduce the complications due to low BMD [17,18,26,27]. Particularly, lattice-designed cages that mimic the web-like structure of native cancellous bone have shown excellent resistance to post-operative cage subsidence [28,29,60]. Conversely, a recent investigation conducted by Rezvani et al. [61] revealed that in spondylodiscitis instrumented fusion, autologous bone graft mixtures exhibited a superior fusion rate alongside a lower mortality rate when compared to the use of titanium cages.

BMD is also significant in guiding surgical techniques and approaches. In patients with lower BMD, a posterior approach may be preferred over an anterior one as it can provide more support and stability to the spine, as described above. Even though cage subsidence is relevant to BMD, that subsidence does not apply to clinical deterioration, so PLIF remains a safe option for treating lumbar degenerative diseases in osteoporotic patients [16]. A larger or more robust implant may also improve fusion rates and decrease the risk of implant failure [4]. BMD scores can also help to guide the choice of bone graft material used in LIF surgeries. In osteoporotic patients, bone substitutes, such as demineralized bone matrix, may be preferred over autograft, which may be osteoporotic as well. BMD can also be used to predict outcomes and potential complications of LIF. The risk of non-union or adjacent segment disease may be higher in patients with lower BMD. Non-union occurs when the bone fails to fuse with the implanted graft or device, resulting in persistent pain and instability. Adjacent segment disease refers to the degeneration of spinal segments adjacent to the site of the LIF surgery. It can occur due to altered biomechanics or increased stress on the adjacent segments. Closer monitoring and follow-up may be necessary in patients with lower BMD to detect and manage these potential complications [4].

BMD can also impact postoperative management and rehabilitation. Patients with lower BMD may require extended periods of immobilization and activity restriction to allow for adequate bone healing and integration of the implanted graft or device. Physical therapy and exercise programs may also need to be modified to account for decreased bone strength and increased fracture risk.

Pharmacological enhancement of LIF

BMD is an essential factor in the success of LIF procedures. Low BMD, as described before, can increase the risk of complications such as implant failure, non-union, and postoperative fracture. Therefore, managing BMD before the procedure is essential to reduce the risk of these complications.

Osteoporosis is widely recognized for its impact on bone quality, primarily due to adverse bone remodeling. Reduced bone quality, assessed through BMD, diminishes the pull-out strength of pedicle screws, and adverse bone remodeling leads to delayed bone fusion [30,31]. Therefore, spine surgeons should consider osteoporosis pharmacological management before performing a LIF procedure. Certain medications such as bisphosphonates, denosumab, romozumab, and parathyroid hormone (PTH) analogs like teriparatide can help improve BMD. These medications work by slowing down bone loss or promoting bone growth.

Bisphosphonates

Bisphosphonates are pyrophosphate analogs that exhibit a strong binding affinity for hydroxyapatite. Studies have demonstrated their ability to decrease biomarkers associated with bone resorption and turnover, such as bone alkaline phosphatase (BAP), beta-C-terminal telopeptide (β-CTX), and N-terminal telopeptide (NTX), elevate BMD and avert fragility fractures [36-38]. Bisphosphonate therapy promotes the apoptosis of osteoclasts and exhibits favorable outcomes compared to control groups, including a reduction in vertebral compression fractures, reduced rate of screw loosening and increased fusion rates [31,39,40].

The studies conducted on bisphosphonate therapy have demonstrated a positive impact. However, Buerba et al. [41] did not find any significant differences in fusion rates and screw loosening between patients who received bisphosphonates and those who did not. It is important to note that the evidence supporting bisphosphonate therapy for spinal fusion is limited, and more research is required to establish its effectiveness.

Teriparatide

Teriparatide is an anabolic recombinant parathyroid hormone (rhPTH-34) that improves BMD and reduces fracture risk [42]. It is a medication that is officially approved for treating osteoporosis in postmenopausal women and men at a higher risk of bone fracture. It is also used to treat osteoporotic patients who have been receiving prolonged and systemic glucocorticoid therapy. In addition, it is prescribed to patients who are under treatment with bisphosphonates, but are suffering from osteoporotic fractures, fragility fractures or have a decreasing BMD [43,44]. Off-label use of teriparatide has been shown to enhance osteoblastic bone formation when administered as a daily subcutaneous injection [45]. Research indicates that administering weekly subcutaneous teriparatide in older women undergoing TLIF/PLIF for lumbar degenerative spine diseases can improve interbody fusion. This results in higher fusion rates when compared to a control group [33]. Weekly teriparatide use was shown to be a predictive factor of intervertebral union six months after PLIF in another study [34]. Although most of the above studies indicate positive results, the literature consists primarily of case reports or case series, which presents a clear risk of bias. It is essential to note that teriparatide is not typically used as a standalone treatment for LIF, and the optimal dosage and administration regimen for clinical application is yet to be determined [46].

Vitamin D

Vitamin D, a hormone essential for calcium absorption and bone mineralization, is positively associated with BMD [47]. A solitary randomized controlled trial exclusively investigating vitamin D3 was identified as well. The findings from this study indicated that vitamin D alone might be effective in enhancing fusion rates among patients undergoing TLIF. However, these rates declined at the 12- and 24-month follow-ups compared to control groups [35].

Denosumab

Denosumab is a human anti-receptor activator of nuclear factor kappa beta (RANKL) monoclonal antibody and has been recently used for its effect on the increase in BMD and the treatment of postmenopausal osteoporosis [48-50]. Ide et al. [22] suggested that combining denosumab with teriparatide may accelerate spinal fusion in patients undergoing PLIF compared to those receiving teriparatide alone. However, denosumab has its own set of adverse effects including hypocalcemia, osteonecrosis of the jaw and atypical femoral fractures [51], limiting its use to each patient’s tolerability. Further research is needed to clarify whether denosumab could have a positive impact on spinal fusion.

Romosozumab

Romosozumab is a humanized monoclonal antibody that binds and inhibits sclerostin, with a dual effect of increasing bone formation and decreasing bone resorption [52,53]. Only one study by Cosman et al. [54] indicates that treating postmenopausal women with osteoporosis with romosozumab for one year reduces the risk of vertebral and clinical fractures compared to a placebo. Although data is limited, romosozumab is expected to effectively promote vertebrae healing due to its proven efficacy in treating osteoporosis.

**Figure 1:**
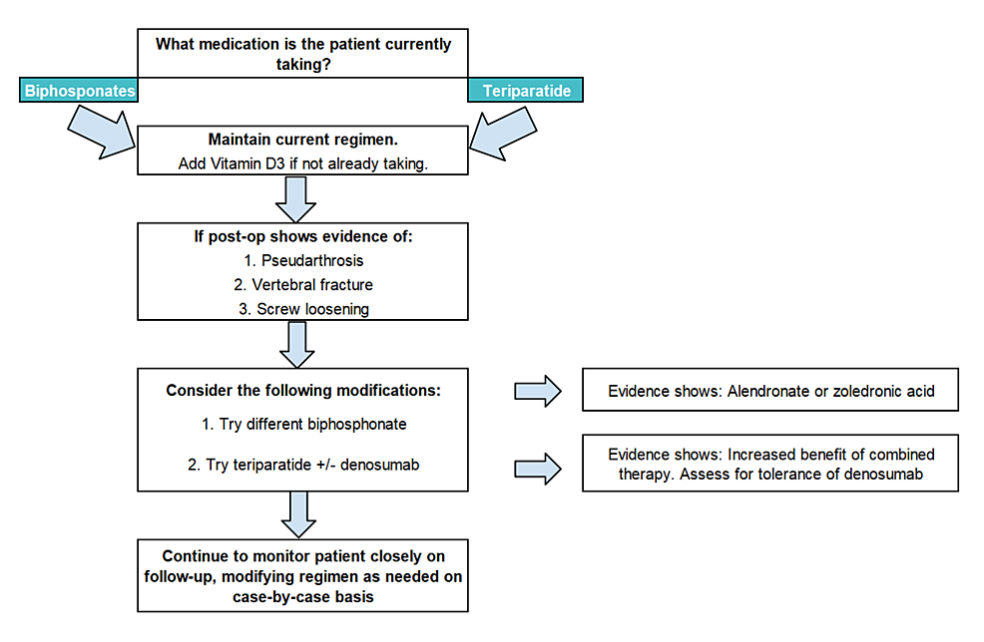
Decision algorithm of pharmacological agents for patients undergoing lumbar interbody fusion (LIF) This figure is a creation of the authors based on Soldozy et al. [32]

While it might be rational for a spine surgeon to consider using bisphosphonates, teriparatide offers superior outcomes in enhancing fusion and preventing complications. Therefore, if pharmacological therapy is initiated, it is advisable to prioritize teriparatide over bisphosphonate therapy unless the patient is already undergoing bisphosphonate treatment. Considering the rare side effects of vitamin D3, it could be administered to all osteoporotic patients, especially those undergoing LIF, as many patients are either deficient or already under treatment. It is of utmost importance that patients on antiosteoporotic treatment are encouraged to maintain postoperatively their current therapeutic regimen, as evidence indicates potential benefits with minimal harm or drawbacks [32]. The decision to support LIF techniques with pharmacological enhancement will depend on several factors, including the patient's comorbidities, the severity of their osteoporosis or other bone-related conditions, and the specific details of their surgical procedure. A proposed decision algorithm is provided in Figure 1 [32].

Biomechanical enhancement in LIF

Biomechanical enhancement in LIF refers to the utilization of various techniques and technologies aimed at improving the mechanical stability and long-term success of the fusion procedure. As previously mentioned, reduced vertebral BMD can make it challenging to perform surgical interbody fusion as the weak bone structure can increase the risk of implant-related complications such as cage subsidence after the procedure. Fusion construct length, selection of screws, choice of bone grafts, preparation methods, utilization of intervertebral prostheses (IVPs), extent and type of neural decompression, and management of neural damage, while considering extraspinal trauma and existing comorbidities, represent significant considerations in lumbar fusion [62,63].

Several strategies are employed to achieve biomechanical enhancement in LIF. Advanced, lattice-designed cages resembling the web-like structure of the native cancellous bone have shown resistance to post-operative subsidence and can be a promising solution [17,55-57]. These implants have an open, porous structure that supports osseointegration and vascularization. They are designed to maximize contact with the apophyseal ring while being optimized for size. They feature a textured surface modification which allows new bone matrix to interdigitate within the crevices and asperities on the roughened surface [17,58]. As a result, a secure bond at the bone-implant interface is formed [59].

Moreover, interbody cage devices can be tailored to match a patient’s BMD T-score by modulating the density and compactness of the pore structure, ensuring optimal load distribution and minimizing stress concentration at the implant-bone interface. These cages are available to support patients across the BMD spectrum, including those with osteoporosis. Three designs are available, each reflecting low-, mid-, and high-density BMD T-scores as classified by DXA. Falowski et al. [17] have developed an interbody fusion device that has a distinct biomechanical profile, which is specific to bone density. This device could be particularly useful in patients who suffer from osteoporosis and need LIF. Further research is recommended to evaluate the clinical efficacy of these design features.

Mo et al. conducted a study comparing the safety and efficacy of cement-augmented pedicle screws to traditional pedicle screw techniques in patients with osteoporotic spines and lumbar degenerative diseases [64]. They observed that the cement-augmented pedicle screw technique proved effective and safe in this population, demonstrating superior fusion rates and fewer instances of pedicle screw loosening. Notably, while no significant differences were observed in fusion rates and pedicle screw loosening rates between the two groups in single-segment patients, the cement-augmented pedicle screw group exhibited improved fusion rates and lower pedicle screw loosening rates in patients with double or multiple segments affected.

In a compelling meta-analysis investigating bone graft selection in instrumented spinal surgery, Tavares et al. discovered that local bone grafts yielded superior overall functional and radiological outcomes compared to alternative bone substitutes [65]. Rezvani et al. also observed that utilizing an autologous bone graft mixture, as opposed to titanium cages, can lead to a notable increase in fusion rates coupled with a lower mortality rate. However, they noted that clinical outcomes may be adversely impacted by neurological symptoms present upon admission, comorbidities, and advanced age [61].

## Conclusions

This review highlights the crucial yet controversial role of BMD in the success of LIF surgery. The available evidence consistently demonstrates that low BMD is a significant risk factor for fusion failure and is associated with an increased risk of complications such as implant migration, implant subsidence, and pseudoarthrosis. Osteoporotic or osteopenic individuals may necessitate additional interventions or extended recovery periods, underscoring the imperative of BMD assessment before LIF surgery. While the efficacy of bisphosphonate therapy remains uncertain for osteoporotic patients undergoing LIF, teriparatide emerges as a preferable pharmacological option. Moreover, the synergistic administration of teriparatide and denosumab holds promise for further augmenting spinal fusion outcomes. The advent of BMD-specific interbody cages presents a potential avenue for enhancing fusion success in spinal surgery, albeit warranting further investigation for comprehensive clinical integration. 
